# BDNF influences neural cue-reactivity to food stimuli and food craving in obesity

**DOI:** 10.1007/s00406-020-01224-w

**Published:** 2020-12-26

**Authors:** Jan Malte Bumb, Patrick Bach, Martin Grosshans, Xenija Wagner, Anne Koopmann, Sabine Vollstädt-Klein, Rilana Schuster, Klaus Wiedemann, Falk Kiefer

**Affiliations:** 1 Department of Addictive Behavior and Addiction Medicine, Medical Faculty Mannheim, Central Institute of Mental Health, Heidelberg University, J5, Mannheim, Germany; 2 Feuerlein Center on Translational Addiction Medicine (FCTS), University of Heidelberg, Heidelberg, Germany; 3 Mannheim Center for Translational Neurosciences (MCTN), Medical Faculty of Mannheim, University of Heidelberg, Mannheim, Germany; 4 Department of Psychiatry and Psychotherapy, University Medical Center, Martinistr, 52, 20246 Hamburg, Germany

**Keywords:** Addiction, BDNF, Craving, Insula, fMRI, Obesity

## Abstract

There is increasing evidence that brain-derived neurotrophic factor (BDNF) impacts on the development of obesity. We are the first to test the hypothesis that BDNF levels might be associated with neural reactivity to food cues in patients suffering from obesity and healthy controls. We assessed visual food cue-induced neural response in 19 obese patients and 20 matched controls using functional magnetic resonance imaging and analyzed the associations between BDNF levels, food cue-reactivity and food craving. Whole-brain analysis in both groups revealed that food cues elicited higher neural activation in clusters of mesolimbic brain areas including the insula (food > neutral). Patients suffering from obesity showed a significant positive correlation between plasma BDNF levels and visual food cue-reactivity in the bilateral insulae. In addition, patients suffering from obesity with positive food cue-induced insula activation also reported significantly higher food craving than those with low cue-reactivity—an effect that was absent in normal weight participants. The present findings implicate that BDNF levels in patients suffering from obesity might be involved in food craving and obesity in humans. This highlights the importance to consider BDNF pathways when investigating obesity and obesity treatment.

**Supplementary Information:**

The online version contains supplementary material available at 10.1007/s00406-020-01224-w.

## Introduction

Worldwide, the prevalence of obesity, which is defined by having a body mass index (BMI) of 30 kg/m^2^ or higher nearly tripled since 1975 [1]. In 2016, more than 1.9 billion adults were overweight or obese [1]. This is clinically relevant since obesity is associated with a significant increase in all-cause mortality [2]. Obesity is also a major risk factor for type 2 diabetes mellitus, cardiovascular disease, hypertension and also influences on the development of osteoarthritis and some forms of cancer, including breast, prostate, liver and colon [3].

Currently available antiobesity treatments include lifestyle interventions, pharmaco- and psychotherapy, bariatric surgery as well as combinations of the different approaches [3]. However, the treatment of obesity is limited by insufficient efficacy and high risk for regaining of weight [3]. The unsatisfactory treatment effects are partly because the current knowledge of the pathophysiology of obesity, and especially on factors that modulate appetitive mechanisms remains elusive.

Etiopathologically, obesity has been linked to genetic and epigenetic factors [3, 4]. Moreover, obesity often results from a positive energy balance, i.e. increased intake of energy-dense foods and a decrease of physical activity [3, 4]. Aside from these well-established factors, there is increasing evidence that obesity shares certain similarities with addictive behaviors [4–7]. In this respect, craving, i.e. a strong urge or desire, which is well-known as a high-risk factor for consumption of or relapse to drugs of abuse, is also commonly reported by patients suffering from obesity [8–10]. In these individuals, the sight or smell of a certain food might trigger a strong desire to eat [11]. According to Boswell et al. [11], the sight or smell of food might act like conditioned stimuli if a subject is frequently exposed to these cues—and if they predict food consumption. By this means, food craving as well as neural reactivity to food cues represent conditioned responses to the conditioned stimuli. Boswell et al. [11] reported that visual food cues (e.g. pictures and videos) were associated with food craving and eating behavior, just as exposure to real food.

While the hypothalamus has been hypothesized to control metabolic drives to eat [4], cortical areas, the basal ganglia, the hippocampus, and the amygdala integrate emotional, cognitive, and executive information [4]. In addition, sensory information (visual, olfactory, and auditory), as well as oral taste are processed in brain areas such as the orbitofrontal, prefrontal, and insular cortex [12]. In particular, the insula holds a unique position since it integrates information on the oral taste and the internal milieu through the vagus nerve [4]. Moreover, the anterior insula is crucial for the generation of self-awareness and consciousness [13]. Since the publication of the milestone paper of Naqvi et al. [14], which reported on the relevance of the insula for maintaining and disruption of tobacco addiction respectively, there is cumulating evidence for the participation of the insula in the development and the maintenance of addictive behavior in general [15]. In this context, the insula and in particular the right insula [16] has been hypothesized to integrate interoceptive signals and transform this information to conscious awareness, preceding the formation of memory and executive functions, which together control motivated behavior and also promote taste learning [17, 18]. Several studies have shown that these processes require and are dependent on BDNF mediated protein synthesis in the insula (for review see Ref. [18]). Moreover, BDNF protein expression in the insula has been hypothesized to facilitate plasticity encoding the hedonic value of certain conditioned stimuli—also in the absence of reinforcement [19]. At the same time, disruption of BDNF protein secretion or signaling may significantly impair memory acquisition, retention, and recall [18].

However, there is a large variance in findings, suggesting that multiple factors might influence cue-reactivity and craving—of which the majority are currently not fully understood. Against this backdrop, studies investigating the basis of food-induced craving in obesity (e.g. Ref. [20, 21]) pointed towards a role of peptides in the pathophysiology of food craving and overeating. It has been stated that brain area-specific BDNF levels might be associated differentially to energy intake and expenditure when investigating normal-weight participants and patients suffering from obesity. In fact, deletions inducing haploinsufficiency of BDNF were suggested to result in severe obesity in mice [22]. Furthermore, food deprivation selectively reduces BDNF mRNA in the ventromedial hypothalamus as well as BDNF protein levels in the dorsal vagal complex in rodents [23] and deficient BDNF signaling makes mice more vulnerable to hyperphagia on high-fat but not on low-fat diets [24]. Altogether, these data highlight a role of BDNF in hedonic eating and in obesity.

Already in 1998 [25], it was stated that in humans peripheral BDNF crosses the blood–brain barrier. A more recent study [26] reported that blood—whole blood and plasma—BDNF levels reflect brain-tissue levels in rats and pigs. Interestingly, pig plasma BDNF levels were comparable to previously reported values in humans [26]. Additionally, positive correlations between whole-blood BDNF levels and hippocampal BDNF levels in rats and between plasma BDNF and hippocampal BDNF in pigs were revealed. Moreover, a significant positive correlation between frontal cortex and hippocampal BDNF levels in mice was demonstrated. To conclude, evidence suggests that peripheral BDNF levels mirror central levels.

We here hypothesize that BDNF might be associated with blood oxygenation level dependent (BOLD) signal changes since BDNF influences energy intake and expenditure through the tropomyosin-related kinase B (TrkB) receptor (for review see Ref. [27]). Activation of the BDNF-TrkB pathway triggers a magnitude of intracellular signaling including dopaminergic, GABAergic, glutamatergic, and muscarinic neurotransmission as well as subsequent activation of protein kinases, transcription factors (e.g., cyclic AMP responsive element binding protein [CREB]) and early genes (c-fos, Arc) [18]. Intriguingly, dopamine D1 neurons are expressed in brain regions known to regulate food intake, including the ventral striatum, and hypothalamic nuclei including the paraventricular and suprachiasmatic nuclei [28], the amygdala and the medial prefrontal cortex (for review see Ref. [27]) as well as the insula (for review see Ref. [18]). In addition, it has been shown that dysfunction of BDNF-TrkB signaling as well as deletion of TrkB receptors from dopamine D1 neurons may result in obesity [28].

To sum up, BDNF mainly influences on energy homeostasis via the TrkB pathway, which again mediates energy balance by modulating synaptic plasticity of certain brain regions, including the insula and mesolimbic (dopaminergic) pathways (for review see Ref. [27]). It seems plausible that these processes are measureable by using fMRI—as demonstrated in a recent study showing that the CREB-BDNF pathway influences on alcohol cue-elicited brain activation in the precuneus, superior parietal lobule, and posterior cingulate in drinkers ADDIN EN.CITE [29].

To the best of our knowledge, the association of BDNF levels and cue-induced brain activation during the presentation of visual food cues has not been studied to date. fMRI and cue-reactivity analyses, as a measure of neural response to e.g. visual cues, are a valuable basis for studying etiological concepts of overweight, obesity and craving for food.

To further elucidate the biological and neural underpinnings of obesity, we investigated the association between BDNF levels and brain response to food stimuli and craving in patients suffering from obesity and normal-weight participants by conducting a prospective case–control fMRI study.

## Experimental procedures

The study population has been described in more detail in a previous study investigating associations between food cue-induced activation in the human reward circle and plasma levels of leptin [21].

### Participants

All participants provided written informed consent and the study was approved by the local ethics committee (ethics committee II—Heidelberg University). Patients suffering from obesity fulfilled the following inclusion criteria: (i) age between 18 and 65 years, (ii) BMI (kg/m^2^) ≥ 30 (accepted threshold for obesity by the World Health Organization), (iii) a waist circumference < 150 cm (limited by scanner diameter), (iv) the capacity to give informed consent, (v) no history or current diagnosis of any psychiatric, neurological, neoplastic or untreated endocrine illnesses (with the exception of nicotine addiction), and (vi) no current intake of any psychoactive or antiobesity medications. Individuals of the control group had to meet all the above-stated inclusion criteria with the exception of having a BMI > 18.5 but < 25, corresponding to the normal weight range defined by the World Health Organization. All participants with a history of surgical interventions in the gastrointestinal system or contraindications to fMRI scanning (e.g. metal implants) were excluded.

Fifty individuals (24 patients suffering from obesity and 26 normal-weight participants) were eligible for analyses and included in the study. BDNF could not be measured in two healthy controls and two obese individuals (lack of material). Moreover, imaging data were unavailable for four healthy controls and three obese individuals due to insufficient data quality (e.g., heavy movement, artefacts). The resulting study population consisted of 19 patients suffering from obesity and 20 healthy, age-matched and sex-matched normal-weight participants.

On the day of testing, all participants received a standardized breakfast of 500 kcal (2093 kJ) 6 h before fMRI scanning. Subsequently, participants completed a series of questionnaires including the Beck Depression Inventory (BDI, [30] and Fagerstroem Test for Nicotine Dependence (FTND [31]). In addition, participants provided demographical data. Before scanning, 30 mL of full blood was drawn from a cubital vein for use in neuroendocrine analyses. Blood samples for the determination of BDNF were drawn immediately prior to scanning.

### BDNF analyses

To ensure that the storage of the blood samples does not influence blood levels of BDNF, the samples were anticoagulated with sodium ethylenediaminetetraacetic acid (1 mg/mL of whole blood) and stored immediately after withdrawal at − 80 °C [32].

Plasma BDNF analyses were performed at the Neurobiological Laboratory of the Department of Psychiatry, University of Hamburg (K.W.) and we used a human BDNF ELISA kit (IBL, Hamburg, Germany). The kit had a detection limit of 0.08 ng/mL for plasma concentrations of BDNF. Intra-assay and inter-assay coefficients of variation were below 10% and 12%, respectively.

### Imaging procedure

#### fMRI food cue-reactivity task

During the imaging session, visual food and neutral stimuli were presented in blocks that were pseudo-randomized. The task included a total of 18 blocks of food stimuli and 12 blocks of neutral stimuli, consisting of a series of 5 food or neutral pictures from the same category each. Food stimuli were further arranged in three categories: salty high-calorie, sweet high-calorie, low-calorie, yielding 6 blocks for each category. All stimuli were shown for 4 s (i.e. 20 s per block). Between each picture stimulus block, participants' food craving was assessed with a visual analogue scale (VAS) that ranged from 0—“very weak” to 100—“very strong”. The exact wording of the craving question was—“How strong are your food cravings now”. The fMRI task lasted a total of 18 min. Food stimuli chosen were rated according to their ability to induce food craving by 44 voluntary participants at our institution [21]. Neutral cues were taken from the International Affective Picture Series [33].

#### fMRI acquisition and pre-processing

Scanning was performed using a 3-T whole-body tomography scanner (MAGNETOM Trio with TIM technology; Siemens). T2*-weighted, echo planar images covering the entire brain were acquired. Imaging parameters were set to repetition time = 2.41 s, echo time = 25 ms, flip angle = 80°, number of slices = 42, slice thickness = 2 mm, voxel-gap = 1 mm, voxel dimensions = 3 × 3 × 3 mm^3^, field of view = 192 × 192 mm^2^, in-plane resolution = 64 × 64. We chose the short echo time and the 30° flip angle to anterior commissure–posterior commissure orientation to minimize susceptibility artefacts. 453 images were acquired per subject. Visual stimuli were presented and craving data recorded using Presentation software (version 9.9, Neurobehavioral Systems Inc.) and MRI-compatible goggles and response pads (MRI Audio/Video Systems; Resonance Technology Inc., CA).

Functional-imaging data were processed and analyzed using SPM5 (pre-processing and individual statistics) and SPM8 (second-level group analyses; Wellcome Department of Cognitive Neurology, London, UK). The first 5 scans were excluded from imaging analyses to avoid any artefacts caused by the effects of magnetic saturation. All images were realigned spatially (movement was considered excessive with > 2 mm translation or > 2° rotation), normalized to a standardized EPI template from MNI (Montreal Neurological Institute, Quebec, Canada), and smoothed using an isotropic Gaussian kernel for group analyses (full width at half maximum: 8 mm).

### Statistical analysis

First-level statistical analyses of imaging data were carried out by modelling the different conditions (salty high-calorie, sweet high-calorie, low-calorie and neutral) as explanatory variables within the context of a general linear model, which also included movement parameters as nuisance variables. Individual contrast images (food cues > neutral cues) were computed for each individual and then included in our second-level analyses. We used a flexible factorial model with group × BDNF levels to assess group differences and group-dependent BDNF effects. Previous work found that nicotine and leptin alter food–cue reactivity [21, 34]. Therefore, the effects of both variables were controlled in the group analyses of imaging data. In addition, to ensure that our group differences were not driven by the clinical measures that differed between our groups, we considered the BDI scores as a covariate in our analyses. To satisfy a family-wise error rate correction of pFWE < 0.05, we determined a combined height (*p* < 0.001) and extent (*k* ≥ 41) threshold by running 10.000 Monte Carlo simulations using AlphaSim as implemented in the Neuroelf analysis package (www.neuroelf.net) [35].

To test associations between neural activation and subjective craving, we extracted the functional food cue-induced activation (contrast: food > neutral) in regions that showed group effects (obese > control) and associations with BDNF scores, i.e. the left and right insula. Region of interest (ROI) masks for the left and right insula were derived by computing the intersection between standardized anatomical masks of the left and right insula from the Wake Forest University PickAtlas (WFU PickAtlas; www.fmri.wfubmc.edu/downloads) and areas in the insulae that showed significant supra-threshold activation in the group analyses. This procedure was chosen to yield greater specificity of results, i.e. only voxels in the areas that were indicated in group analyses are considered. Mean functional activation (contrast: food > neutral) was extracted from the left and right insula ROI using a custom SPM toolbox that is described in detail by Reinhard and colleagues [36]. Sample characteristics, correlations between functional brain activation and subjective craving were all analyzed using the Statistical Package for the Social Sciences (SPSS, IBM Corp., Somers, NY, USA) version 21.0. Independent sample *t*-tests were performed to test differences between obese and non-obese participants. In addition, repeated measures multivariate analyses with stimulus category (food, neutral) and group (non-obese, obese) were applied to investigate effects on craving during the scanning session. Due to the fact that: (i) previous research showed a positive association between leptin levels and subjective food-craving [21] and (ii) current results show a difference between both experimental groups, separate univariate analyses, controlling for leptin, were used to determine associations between cue-reactivity in the left and right insulae (dichotomized into positive ≥ 0 and negative < 0) and food craving.

## Results

### Sample characteristics, craving data and hormonal analyses

Demographic variables, obesity status and clinical data (i.e., BDI, FTND) are summarized in Table 1. The mean age of the sample was 40.1 (SD = 11.2) years, with no significant difference between patients suffering from obesity and normal-weight participants (*p* = 0.752). Blood samples showed a mean BDNF level of 115.9 ng/mL (SD = 24.5) and no difference between obese and normal-weight participants (*p* = 0.968). Both groups differed significantly with regard to BMI (*t*(37) = 10.86, *p* < 0.001), waist circumference (*t*(37) = 13.05, *p* < 0.001) and mean leptin levels (*t*(37) = 6.23, *p* < 0.001).

**Table 1:** Demographic and clinical data

	Group		Statistics	*p*
	Obese (*n* = 19)	Non-obese (*n* = 20)		
Demographical variables				
Age (years)	39.6 (11.2)	40.8 (11.4)	*t*_(37)_ = 0.318	0.752
Sex (female:male)	13:6	14:6	*χ*^2^_(1)_ = 0.011	0.915
BMI (kg/m^2^)	36.7 (5.9)	21.9 (1.6)	*t*_(37)_ = 10.861	** < 0.001**
Waist circumference (cm)	115.5 (11.8)	75.1 (7.1)	*t*_(37)_ = 13.045	** < 0.001**
Smoker (yes/no)	5:14	5:15	*χ*^2^_(1)_ = 0.009	0.925
Peptides				
BDNF (ng/mL)	115.8 (25.0)	116.1 (24.7)	*t*_(37)_ = 0.040	0.968
Leptin (ng/dL)	26.9 (13.8)	6.2 (5.4)	*t*_(37)_ = 6.245	** < 0.001**
Clinical scales				
FTND (sum score)	0.6 (1.5)	0.8 (1.9)	*t*_(37)_ = 0.397	0.694
BDI (sum score)	7.9 (7.2)	2.8 (3.3)	*t*_(37)_ = 2.886	**0.006**
Cue-induced craving				
Food cues	55.4 (23.2)	53.3 (18.2	*t*_(37)_ = 0.320	0.751
Neutral cues	23.6 (24.9)	27.1 (19.2)	*t*_(37)_ = 0.501	0.618

*BDI *Beck Depression Inventory, *FTND *Fagerstroem Test for Nicotine Dependence

Patients suffering from obesity showed a higher BDI than the normal-weight participants (sum score 7.7 vs 2.8; *p* = 0.006), the frequency of smokers and the FTND sum scores did not differ between both experimental groups (*p*_min_ = 0.18).

### fMRI food cue-induced brain activation

Whole-brain analyses show a significant main effect of stimulus category (food > neutral) on brain response in patients suffering from obesity, such that food cues elicited higher brain activation in a cluster of brain areas that included the insula, the orbitofrontal cortex, the middle and posterior cingulate cortex, the superior, middle and inferior occipital gyri, the superior, middle and inferior temporal gyri, the cuneus and the fusiform gyrus [see Table 2(a)]. Normal weight participants displayed higher brain activation to food cues, compared to neutral cues in several brain areas, including the insula, the orbitofrontal cortex, the cuneus, the fusiform gyrus, the cerebellum and the lingual gyrus [see Table 2(b)]. We found higher food cue-induced activation in obese individuals in the inferior frontal operculum and inferior frontal triangular gyrus [see Table 2(d)], while normal-weight participants had no higher cue-induced activation in any brain area [see Table 2(e)].

**Table 2:** Brain areas that showed higher activation to food cues compared to neutral cues in (a) obese individuals and (b) normal-weight participants and (c) brain regions that show a significant interaction between BDNF levels and group status (obese vs. non-obese). Obese individuals had higher cue-reactivity in parts of the inferior frontal gyrus (d), while (e) the control group showed no significant higher activation in any brain area (contrast: “food—neutral”, *n* = 39, combined voxel-wise—[*p* < 0.001] and cluster-extent-threshold [*k* > 41 voxel], corresponding to p_FWE_ < 0.05)

Side	Lobe	Brain regions	Cluster size	MNI Coordinates (*x*, *y*, *z*)			*t*_max_
*Within group*							
(a) Obese individuals (*n* = 19)—contrast: Food > Neutral							
L and R	Occipital, Temporal	Superior, Middle and Inferior Occipital Gyrus, Superior, Middle and Inferior Temporal Gyrus, Cuneus, Fusiform Gyrus, Cerebellum, Lingual Gyrus	7221	10	− 90	0	11.28
R	Frontal	Superior, Middle and Inferior Frontal Gyrus	345	50	48	10	5.97
L	Frontal	Middle and Inferior Frontal Gyrus	228	− 42	42	12	4.79
R	Frontal	Orbitofrontal Cortex, Middle Frontal Gyrus	116	42	56	− 8	6.47
R	Occipital	Superior and Middle Occipital Gyrus, Angular Gyrus	113	30	− 62	36	5.49
R	Temporal	Inferior Temporal Gyrus, Fusiform Gyrus	108	54	− 60	− 16	5.28
L		Precentral Gyrus	103	− 42	− 2	28	4.57
R		Cerebellum	74	38	− 78	− 40	5.56
L		Insula	54	− 36	26	0	4.24
L and R		Middle and Posterior cingulate cortex	53	− 2	− 32	26	4.71
L		Insula	53	− 38	2	− 8	4.50
(b) Non-obese individuals (*n* = 20)—ontrast: Food > Neutral							
L and R	Occipital	Superior, Middle and Inferior Occipital Gyrus, Cuneus, Fusiform Gyrus, Cerebellum, Lingual Gyrus	3824	− 16	− 102	6	8.28
R		Cerebellum	137	42	− 58	− 38	5.63
L	Frontal	Orbitofrontal Cortex, Insula, Middle Frontal Gyrus	133	− 26	36	− 14	4.97
R	Frontal	Orbitofrontal Cortex, Rectus, Insula	123	22	26	− 18	6.06
L	Frontal	Superior Medial Frontal Gyrus	55	− 8	60	4	5.40
*Group differences*							
(c) Interaction BDNF levels × group (*n* = 39)							
L		Insula (64% of cluster), Rolandic Operculum	168	− 42	− 4	8	6.02
R		Insula (21% of cluster), Rolandic Operculum	121	42	− 18	24	4.69
L	Frontal	Inferior Frontal Gyrus	110	− 34	24	16	4.58
R	Frontal	Inferior Frontal Gyrus, Postcentral Gyrus	89	40	0	24	4.86
R	Frontal	Superior and Middle Frontal Gyrus	47	− 18	24	42	4.42
(d) Obese > Control group							
L	Frontal	Inferior Frontal Operculum, Inferior Frontal Triangular Gyrus	58	− 48	20	12	3.99
(e) Control group > Obese							
–	––	–	–	–	–	–	–

**Fig. 1:**
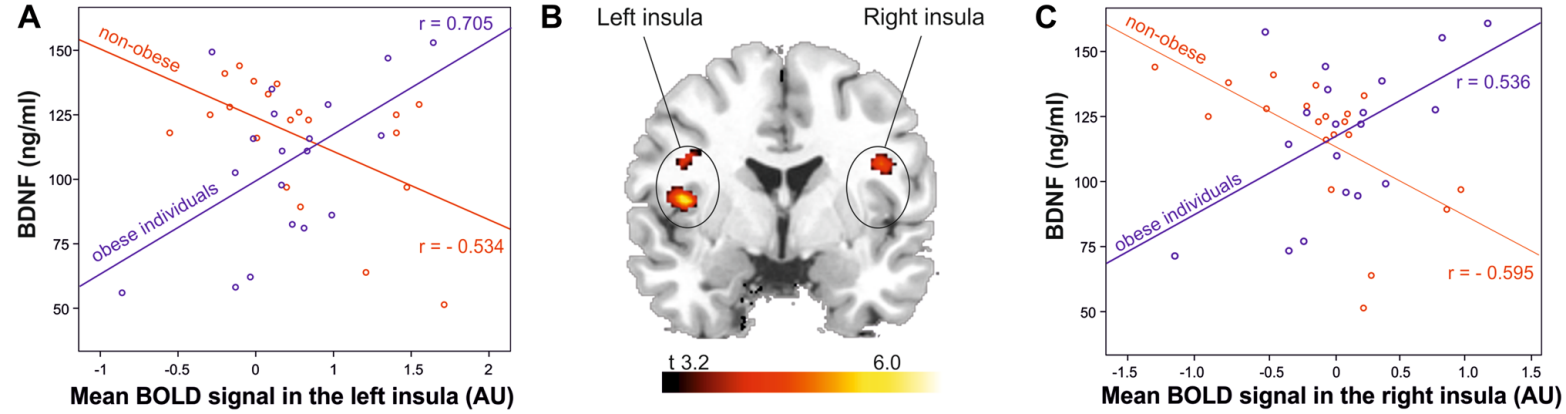
Scatterplots depicting the associations between BDNF levels and mean functional activation in **a** the left insula (*r*_obese_ = 0.705, *p* < 0.001, *r*_normal-weight_ = − 0.534, *p* = 0.008) and **c** the right insula (*r*_obese_ = 0.536, *p* = 0.009, *r*_normal-weight_ = − 0.595, *p* = 0.003) for obese individuals (blue lines and dots) and normal-weight participants (red lines and dots). Depiction of **b** the higher correlation of left and right insula activation with BDNF levels for obese relative to non-obese individuals (contrast: “food—neutral”, *n* = 39, height-threshold: *p* < 0.001, extent-threshold: cluster size ≥ 41 voxel, corresponding to pFWE < 0.05)

In addition, the results demonstrate a significant interaction between BDNF levels and group status (obese vs. non-obese). In fact, patients suffering from obesity and having higher BDNF levels displayed a higher correlation between food-cue induced activation and cue-reactivity in the left and right insula, the superior temporal gyrus, the postcentral gyrus, the inferior frontal gyrus and the rolandic operculum [see Table 2(c)]. Figure 1 depicts the correlation between BDNF levels and food cue-reactivity in the left and right insula in patients suffering from obesity (*r*_Left_ = 0.705, *p* < 0.001; *r*_Right_ = 0.536, *p* = 0.009) and in non-obese group (*r*_Left_ = − 0.534, *p* = 0.008; *r*_Right_ = − 0.595, *p* = 0.003). The correlation coefficients were significantly higher in the groups of patients suffering from obesity compared to the non-obese sample (Fisher’s *Z*_left insula_ = 4.229, *p* < 0.001 and *Z*_right insula_ = 3.686, *p* < 0.001). To further validate the results, we performed additional analyses including food craving as an additional covariate in the statistical fMRI models investigating the interaction between BDNF levels and group status. Results of the analyses replicated the significant interaction effect between BDNF levels and study group on food cue-induced activation in the left and right insula. The results of the additional analyses are depicted in Supplementary Table S1.

### Effects on food craving

**Fig. 2:**
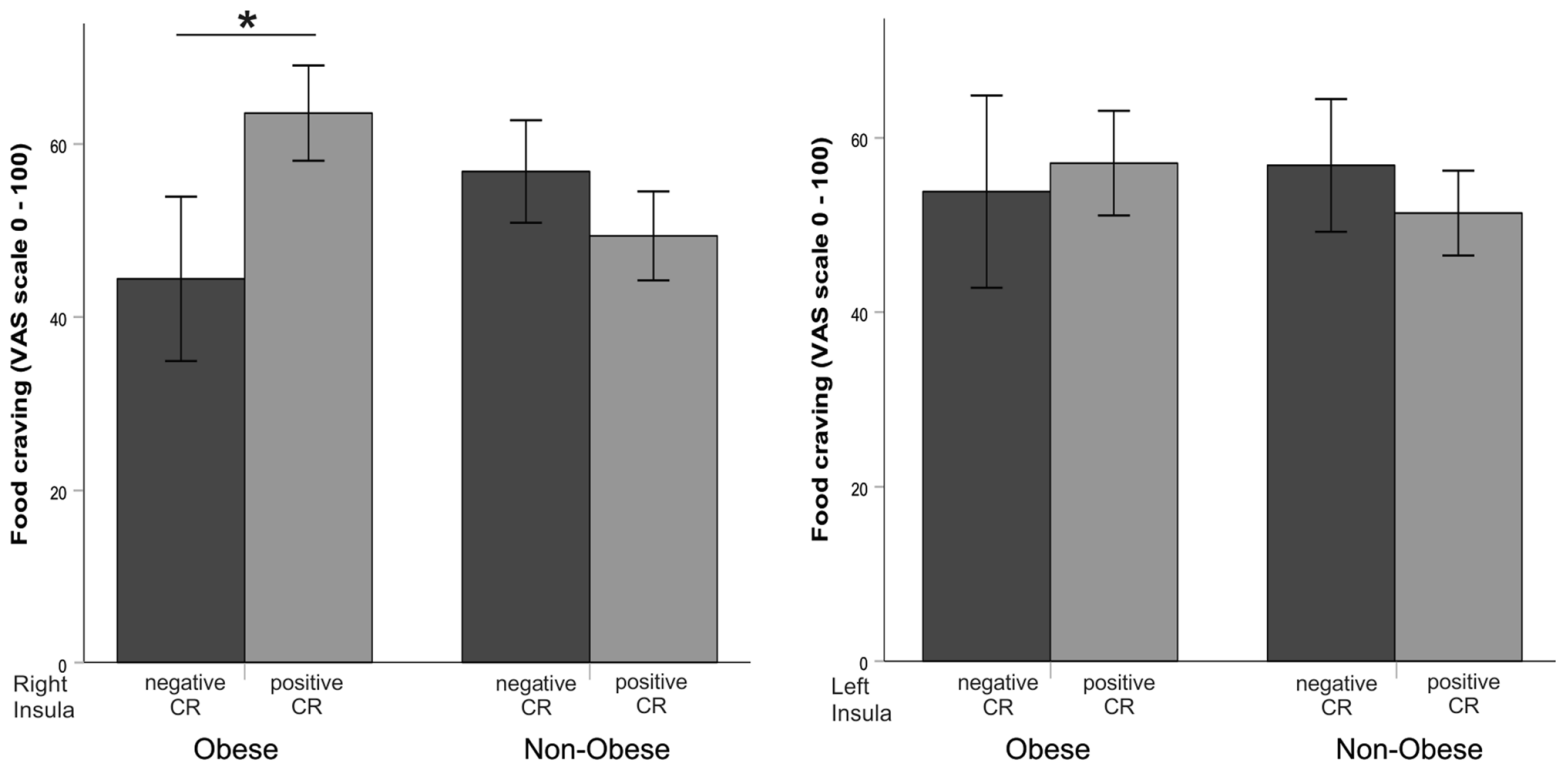
Illustration of the higher food craving [visual analogue scale (VAS)] in patients suffering from obesity with positive cue-reactivity (CR) [mean activation (contrast: food – neutral) > 0] in the right insula (mean = 66.4, SE = 6.9), compared to those with negative cue-reactivity (CR) [mean activation (contrast: food – neutral) ≤ 0] in this area (mean = 42.3, SE = 7.9, *F*_(1,19)_ = 5.204, *p* = 0.048*). Non-obese individuals with positive (mean = 56.0, SE = 8.7) and negative cue-reactivity (CR) (mean = 55.0, SE = 6.3) reported similar food-craving (*F*_(1,20)_ = 0.132, *p* = 0.724)

During the scanning session, food cues (mean = 54.3, SD = 20.6) elicited higher craving than neutral cues (mean = 25.4, SD = 21.9, *F*_(1,21)_ = 41.154, *p* < 0.001). In the group of patients suffering from obesity *n* = 11 participants showed a positive food cue-reactivity in the right insula (i.e. brain activation > 0 for the difference contrast “food vs. neutral”) and *n* = 9 in the left insula. In the normal-weight group, *n* = 9 participants showed a positive food cue-reactivity in the right insula and *n* = 8 displayed a positive food cue-reactivity in the left insula. We observed no significant main effect on the group with regards to subjective food craving during the fMRI session (*F*_(1,21)_ = 0.17, *p* = 0.898, see Table 1), but further univariate analyses in both groups showed that patients suffering from obesity with positive cue-induced right insula activation report higher food craving (mean = 66.4, SE = 6.9) than those with negative cue-reactivity (mean = 42.3, SE = 7.9, *F*_(1,19)_ = 5.204, *p* = 0.048, see Fig. 2), while the normal-weight participants did not show an association between insula activation and extent of food craving (mean_Positive CR_ = 56.0, SE_Positive CR_ = 8.7, mean_Negative CR_ = 55.0, SE_Negative CR_ = 6.3, *F*_(1,20)_ = 0.132, *p* = 0.724). There was no significant difference in the extent of subjective craving between participants with positive vs. negative food cue-reactivity in the left insula, neither in the group of patients suffering from obesity (*F*_(1,20)_ = 0.500, *p* = 0.496), nor in the normal weight group (*F*_(1,19)_ = 0.130, *p* = 0.913, see Fig. 2). We performed additional analyses applying general linear models (GLM) with food craving as a dependent variable, group as a fixed factor and food cue-reactivity in the left and right insulae as covariates of interest, modelling main effects and interactions between cue-reactivity and group status. The models approximated significance (*p*_min_ = 0.063), but failed to surpass the predefined threshold of *p* < 0.05. BDNF levels did not significantly correlate with food-craving during the scanning session, neither across all subjects nor within each experimental group (*p*_min_ = 0.21).

## Discussion

We are the first to show that BDNF is differentially associated with brain responses to food cues in patients suffering from obesity and normal-weight participants.

In the present study, normal-weight participants showing higher BDNF levels were characterized by lower visual food cue-reactivity in the insula. The reported findings implicate that in a physiological and healthy system BDNF might operate by reducing cue-reactivity, which again might prevent craving for food, overeating and obesity. Actually, normal-weight participants did not show a significant association between insula activation and the extent of food craving. Although it might not be intuitive that peripheral BDNF levels are associated with brain response to visual cues, it has been shown that the CREB-BDNF pathway was significantly associated with activation in the precuneus, superior parietal lobule, and posterior cingulate in drinkers suffering from severe alcohol addiction [29]. In the context of energy homoeostasis, BDNF has been linked to both induction of satiety and increasing energy expenditure. It has been reported that food deprivation selectively reduces levels of BDNF mRNA in the mouse ventromedial hypothalamus and that ventromedial hypothalamus neurons might produce BDNF to suppress appetite (for extensive review please see Ref. [27]). In line with this, selective deletion of the BDNF gene in the ventromedial hypothalamus caused moderate hyperphagic obesity [37], while deletion of the BDNF gene in CaMKIIα-expressing neurons in mice resulted in severe obesity. Furthermore, Lapchak et al. [38] showed in rats that chronic intracerebroventricular infusion of recombinant human BDNF attenuated weight gain by suppressing appetite. Accordingly, deletion of the BDNF gene in the paraventricular hypothalamus of mice resulted in obesity by suppressing locomotor activity and thermogenesis [39] while BDNF administration in the ventromedial and paraventricular hypothalamus has been shown to increase metabolic rate in rats [40, 41] and to induce thermogenesis in the brown adipose tissue by facilitating the turnover of norepinephrine in rodents [42]. Obviously, all these mechanisms ultimately may result in weight loss. Taken together, the described results suggest that BDNF might act anorexigenic [27].

The present study also shows that in both groups, visual food cues elicited higher brain activation in a cluster of brain areas including the insula. This is in line with several previous studies [7, 20, 43]. Moreover, this finding validates the fMRI paradigm since mainly the posterior part of the insula receives ascending gustatory and gastrointestinal sensations while the anterior part conveys this information to other brain regions. This part integrates bottom-up and top-down information, i.e., the gustatory state and the rewarding properties of food [16, 44]. Intriguingly, visual food cues elicited higher insula activation in patients suffering from obesity and higher BDNF levels in patients suffering from obesity were associated with higher cue-reactivity in the bilateral insulae if compared to non-obese participants. The directionally opposing associations in normal-weight participants and patients suffering from obesity support an impaired hedonic food regulation in patients suffering from obesity (Fig. 1). Based on the hypothesis that BDNF acts as an anorexigenic agent, it seems counterintuitive that higher BDNF levels were associated with higher visual food cue-reactivity in patients suffering from obesity. However, it might be hypothesized that due to a reduction in BDNF sensitivity in patients suffering from obesity, higher BDNF levels are less likely to prevent food intake, which in turn might be preceded and reinforced by higher visual food cue-reactivity. Therefore, increased levels of BDNF may represent a compensatory mechanism to counteract positive energy balance by suppressing appetite and increasing energy expenditure. This hypothesis is mirrored by studies investigating peripheral BDNF levels in eating disorders that provide evidence that higher BDNF levels in eating disorders might represent an illness-associated and acquired resistance to BDNF with respect to its anorexigenic properties [45]. Therefore, it might be hypothesized that the described anorexigenic feedback mechanism associated with BDNF signaling is attenuated in patients suffering from obesity, comparable with the insulin or leptin resistance as a consequence of long-lasting overeating in patients suffering from obesity [3, 46]. In line with this, Bariohay et al. [23] showed that BDNF infusion in the dorsal vagal complex, which integrates satiety signals from peripheral fat stores, induced anorexia and weight loss. Intriguingly, the ability of BDNF to act anorexigenic in the dorsal vagal complex decreased over a 14-day infusion period. This might add evidence to the above hypothesized “desensitization theory”. Also in support of this, it has been shown that leptin induces the expression of BDNF in the dorsal vagal complex and the ventromedial hypothalamus (for review see Ref. [47]) and it has been repeatedly shown that patients suffering from obesity are characterized by both high leptin levels as well as a leptin resistance.

In addition, a recent meta-analysis [11] highlighted that food cue-reactivity might predict craving for food, overeating and weight gain by activating mesolimbic dopamine pathways (e.g. ventral tegmental area, nucleus accumbens, amygdala, striatum) [48] even irrespective of hunger signals, overruling hormones and energy balance regulation [49], which might lead to continued food intake and poor treatment outcomes [49]. In rodents, depletion of BDNF in the ventral tegmental area led to excessive intake of palatable foods. Noteworthy, peripheral administration of a D1 receptor agonist normalized ingestion of palatable foods, indicating that BDNF influences on dopamine secretion in the ventral tegmental area. Consequently, it might be hypothesized that BDNF plays a role in mediating hedonic reward (for review see Ref. [47]).

Against this backdrop, it has also been reported that craving for food [50] and an attentional bias towards food cues [51] promote overeating and obesity. Additionally, participants with excess weight showed increased functional connectivity between the ventral striatum and the medial prefrontal and parietal cortices and between the dorsal striatum and the somatosensory cortex while dorsal striatum connectivity correlated with food craving and predicted weight gain [52]. Our results are in line with these findings by demonstrating a high cue-reactivity in the right insula to be associated with craving for food in patients suffering from obesity (Fig. 2). This is also corroborated by findings of Wonderlich et al. [53], demonstrating that women with greater activation in the caudate, insula and amygdala suffered from significant increases in craving for food prior to binge eating. The authors concluded that neural response to food cues may influence the daily experience of craving for food and eating habits [53]. Very recently, a further study [43] aimed to test the link between visceral fat and the functional connectivity of the middle-dorsal insula and the rostral insula showed that higher visceral adiposity was associated with decreased connectivity between the middle-dorsal insula and a cluster involving the hypothalamus and the bed nucleus of the stria terminalis as well as between the rostral insula and the right amygdala. Decreased connectivity in this network was associated with greater food craving, a relation mediated by visceral adiposity [43].

The above-described results point towards altered interoceptive feedback loops in both obesity and eating disorders, such as anorexia and bulimia nervosa [54] as well as binge eating [53].

However, it should be noted that, in the present study, the significant difference in the extent of subjective craving between obese patients with positive vs. negative food cue-reactivity could be demonstrated for the right, but not the left insula and only when comparing dichotomous groups (i.e. positive vs. negative food cue-reactivity) and not when testing main effects and interactions of food cue-induced brain activation in the left and right insula as continuous variables in the framework of a general linear model. Even though we observed a significant main effect of food-cue reactivity in the insula in the whole-brain analyses, only about a half of the obese and non-obese patients showed positive mean brain activation in the left and right insula ROI values. Indeed, higher mean activation in the whole group analysis does not imply that the majority of participants will depict a positive BOLD response when considering the mean value over all voxels in the functional insula mask. Using functional ROI masks provides a measure, which shows close associations with clinical measures [36]. The pattern that about 50% of the sample show a positive cue-reactivity has also been observed in previous cue-reactivity studies and hence is not unexpected [55]. Additionally, results did not survive conservative correction for multiple testing. Against this background, the limited size of the sample and the fact that we further conducted subgroup analyses should be taken into account. Hence, current results should be viewed as exploratory and await further validation and replication. Still, current results are in line with previous studies, supporting their plausibility.

It has to be emphasized that in the present study, no group difference between patients suffering from obesity and normal-weight participants with respect to food craving, as well as BDNF levels were revealed. We hypothesize that activation changes on the level of brain networks might not be consciously accessible—as it has been reported elsewhere for alcohol cue reactivity [56]. Additionally, patients suffering from obesity might have underreported their craving due to social desirability. Finally, it might be speculated that the visual food cues chosen in this study were also attractive and appealing for normal-weight participants. Moreover, our finding that BDNF levels do not differ between patients suffering from obesity and normal-weight participants mirrors some [57, 58] but not all previous studies showing either higher BDNF levels [46, 59] or lower levels [60, 61] in patients suffering from obesity, if compared to normal-weight participants. In addition, Monteleone et al. [62] reported significantly reduced serum BDNF levels in underweight patients with anorexia nervosa and in normal-weight patients with bulimia nervosa. However, serum BDNF levels did not differ between overweight patients with binge eating disorder and healthy controls. Finally, it has been reported that a common single nucleotide polymorphism [63, 64] (up to 30% methionine carriers in European [64]), that results in a valine to methionine substitution (in the region of exon IX of the BDNF gene) is related to lower BDNF levels [65], which again has been associated with BMI [66] and eating disorders [67]. Since we did not control for BDNF val66met polymorphism, we cannot rule out that an unequal distribution of that polymorphism between the groups might have influenced the results.

A limitation of the present study is that it cannot resolve the degree to which time of measurement (time of day) or the way that special food may have affected BDNF levels. However, we tried to standardize these aspects by ensuring that on the day of testing, all participants received a standardized breakfast of 500 kcal (2093 kJ) 6 h before fMRI scanning and MRI scanning was scheduled around midday.

## Conclusion

The present study revealed a significant positive correlation between plasma BDNF levels and visual food cue-reactivity in the bilateral insulae in patients suffering from obesity. In addition, obese patients with positive food cue-induced insula activation also reported significantly higher food craving than those with low cue-reactivity—an effect that was absent in normal-weight participants. Our results strengthen the rationale to develop treatments addressing the BDNF pathways [38] to enrich the current multi-modal anti-obesity treatment options taking into account the partly redundant and complex mechanisms underlying the regulation of energy intake and expenditure [4].

## Supplementary Information

Below is the link to the electronic supplementary material.

Supplementary file1 (DOCX 51 KB)
