# Supplementary material for: BDNF influences neural cue-reactivity to food stimuli and food craving in obesity
Source: Eur Arch Psychiatry Clin Neurosci. 2020 Dec 26;271(5):963–74. doi: 10.1007/s00406-020-01224-w (PMC8236045; doi:10.1007/s00406-020-01224-w)
Supplement: Supplementary file 1 — Supplementary file1 (DOCX 51 KB) [file 406_2020_1224_MOESM1_ESM.docx]

Supplementary Table S1. Flexible factorial analyses show (a) a significant interaction between BDNF levels and group status (obese vs. non-obese), while (b & c) no significant main effect of group was found comparing brain activation between obese and non-obese individuals (contrast: “food - neutral”, *n* = 39, combined voxel-wise- [*p* < .001] and cluster-extent-threshold [k > 41 voxel], corresponding to *p*_FWE_ < .05, covariates: BDNF levels, leptin levels, Beck Depression Inventory (BDI) Scores, Fagerstroem Test for Nicotine Dependence (FNTD) Scores, subjective food craving).

| **Side** | **Lobe** | **Brain Regions** | **Cluster Size** | **MNI Coordinates**  **(x, y, z)** | | | ***t*_max_** |
| --- | --- | --- | --- | --- | --- | --- | --- |
|  |  |  |  |  |  |  |  |
|  |  |  |  |  |  |  |  |
| **Group differences** | | | | | | | |
| **(a) Interaction BDNF levels x group (n = 39)** | | | | | | | |
| R |  | **Insula** (17% of cluster), Rolandic Operculum, Precentral Gyrus | 269 | 44 | -18 | 28 | 4.84 |
| L |  | **Insula** (66% of cluster), Rolandic Operculum | 142 | -42 | -4 | 8 | 5.79 |
| L | Frontal | Inferior Frontal Gyrus | 84 | -34 | 24 | 16 | 4.44 |
| R | Frontal | Superior and Middle Frontal Gyrus | 83 | -18 | 24 | 42 | 5.46 |
| L & R | Frontal | Superior Medial Frontal Gyrus | 80 | -14 | 44 | 8 | 4.03 |
| **b) Obese > Control group** | | |  |  |  |  |  |
| - | - | - | - | - | - | - | - |
|  |  |  |  |  |  |  |  |

| **c) Control group > Obese** | | | | | | | |
| --- | --- | --- | --- | --- | --- | --- | --- |
| - | - | - | - | - | - | - | - |
